# Clinical physiology of circadian rhythms: A systematic and hierarchized content analysis of circadian questionnaires

**DOI:** 10.1016/j.ijchp.2025.100563

**Published:** 2025-04-04

**Authors:** Julien Coelho, Vincent P. Martin, Christophe Gauld, Emmanuel d'Incau, Pierre-Alexis Geoffroy, Patrice Bourgin, Pierre Philip, Jacques Taillard, Jean-Arthur Micoulaud-Franchi

**Affiliations:** a Univ Bordeaux, SANPSY, CNRS, UMR 6033, Hôpital Pellegrin, Place Amélie Raba Léon, F-33000, Bordeaux, France; b Service Universitaire de Médecine du Sommeil, CHU de Bordeaux, Place Amélie Raba Léon, F-33000, Bordeaux, France; c Institut des Sciences Cognitives Marc Jeannerod, UMR 5229 CNRS and Université Claude Bernard Lyon 1, Lyon, France; d Département de Psychiatrie et D'addictologie, AP-HP, GHU Paris Nord, DMU Neurosciences, Hopital Bichat-Claude Bernard, 75018, Paris, France; e GHU Paris-Psychiatry & Neurosciences, 1 Rue Cabanis, Université de Paris, NeuroDiderot, Inserm, 75019, Paris, France; f CIRCSom (International Research Center for ChronoSomnology) & Sleep Disorders Center, Strasbourg University Hospital, 1 place de l'hôpital, F-67000, Strasbourg, France; g Institute for Cellular and Integrative Neurosciences, CNRS UPR 3212 & Strasbourg University, 8 Allée du Général Rouvillois, F-67000, Strasbourg, France

**Keywords:** Sleep, Circadian rhythm, Regularity, Self-report questionnaires, Systematic review

## Abstract

Circadian rhythms are near-24 h patterns of physiology and behavior associated with several physical and mental health outcomes. Self-report questionnaires are routinely used and practical tools to assess circadian rhythms. However, the extent to which these questionnaires capture the relevant parameters and can be used interchangeably is unknown. We investigated different types of circadian manifestations using 14 circadian self-report questionnaires for adults. A systematic and hierarchical content analysis was combined with a visualization method. Jaccard indices were calculated to quantify the degree to which the questionnaires overlapped. Content analysis revealed 40 distinct manifestations, which we classified into five dimensions (“circadian phase,” “circadian amplitude and stability,” “nycthemeral timing,” “nycthemeral regularity,” and “circadian complaints"). The average Jaccard index was 0.150, indicating very weak content overlap. None of the 14 questionnaires explored all five dimensions. The Composite Scale of Morningness and the Morningness-Eveningness Questionnaire exhibited greater, but still limited, overlap with the other questionnaires (Jaccard indices of 0.255 and 0.251, respectively), and are the best instruments for assessing the circadian phase. Nycthemeral timing, which must be analyzed to measure the circadian misalignment in clinical and research settings, is only explored by the Munich Chronotype Questionnaire, but that instrument does not evaluate circadian amplitude and stability and only partially assesses nycthemeral regularity. Based on our preliminary analysis, we make recommendations regarding the circumstances in which some circadian questionnaires could prove more useful than the others. The results might also aid the definition and investigation of circadian health at the crossroads of physiology and behavior.

## Introduction

Circadian rhythms are near-24 h physiological (e.g., hormone secretion and body temperature) and behavioral (e.g., eating and sleep) patterns generated by a biological clock and which persist even in conditions of temporal isolation (i.e., in the absence of all external time cues) (Vetter, 2020). These rhythms independently influence physical health (Honkalampi et al., 2021; Vitale & Weydahl, 2017), mental health (Adan et al., 2012; Bauducco et al., 2020), and self-reported morbidity (Taillard et al., 2001). For instance, individuals with an evening preference (eveningness) report more unhealthy behaviors, including increased tobacco and alcohol use, poorer sleep and mental health, and a greater likelihood of being overweight and having diabetes. By contrast, individuals with a morning preference (morningness) show greater resilience to adversity and more optimism, academic success, and adaptability in the workplace (Partonen, 2015). Therefore, assessing circadian rhythms is of great interest in both clinical and research settings.

Objective measures such as the dim-light melatonin onset test and endogenous circadian period (tau; measured via the forced desynchrony protocol) provide accurate and specific assessments of endogenous circadian rhythms (Burgess & Eastman, 2008; Pandi-Perumal et al., 2007). However, they require specialized laboratory equipment and restrictive procedures. Thus, subjective measures of circadian rhythms based on validated self-report questionnaires, which are easier to use, have been widely preferred in clinical and research settings (Levandovski et al., 2013).

The three most cited and utilized self-report questionnaires assessing circadian rhythms are the Morningness-Eveningness Questionnaire (MEQ), the Munich ChronoType Questionnaire (MCTQ), and the Composite Scale of Morningness (CSM) (Horne & Ostberg, 1976; Roenneberg et al., 2003; Smith et al., 1989). These validated instruments have good psychometric properties and have been extensively translated (Adan et al., 2012; Di Milia et al., 2013; Levandovski et al., 2013). The MEQ was even recommended for the evaluation and management of circadian sleep–wake rhythm disorders (CSWRDs) classified according to the International Classification of Sleep Disorders, Third Edition (ICSD-3) and the American Academy of Sleep Medicine guidelines (American Academy of Sleep Medicine, 2014; Morgenthaler et al., 2007). Several other self-report questionnaires have also been developed and evaluated (Byrne et al., 2017; Di Milia et al., 2004, 2011; Dosseville et al., 2013; Folkard et al., 1979; Ogińska, 2011; Ottoni et al., 2011; Putilov et al., 2021; Torsvall & Akerstedt, 1980)

An important issue to consider is whether the above-described tools assess different dimensions of circadian rhythms or capture all of the relevant parameters such that they can be used interchangeably. A cursory glance at the items included in some of the available circadian questionnaires suggests some variability in their content. For instance, the MEQ asks individuals about their preferred time to work (“Suppose that you can choose your own work hours…which five consecutive hours would you select?”) (Horne & Ostberg, 1976), while no such item is included in the MCTQ or the CSM (Roenneberg et al., 2003; Smith et al., 1989). Conversely, the MCTQ investigates bedtime during workdays (“During workdays, I go to bed at _________ o'clock.”) (Roenneberg et al., 2003) and the CSM examines adaptability to an early awake time (“If you always had to rise at 6:00 a.m., what do you think it would be like?”) (Smith et al., 1989) but there are no such items in the MEQ (Horne & Ostberg, 1976). Based on an analysis of several validated self-report questionnaires, (Adan et al., 2012) concluded that “the literature has not explored the inter-relationship between the various circadian rhythm parameters.” Similarly, (Levandovski et al., 2013) found that the three most commonly used questionnaires (the MEQ, MCTQ, and CSM) “evaluate different aspects of circadian typology.” They suggested that in assessing phase preferences, the MEQ and CSM rather measure the endogenous circadian rhythm, while the MCTQ assesses circadian rhythm in more ecological conditions under environmental and behavioral influences. More recently, (Putilov, 2017) pointed to “a disagreement on both the number and content of scales required for multidimensional self-assessment of chronobiological differences." This heterogeneity in content means that the results obtained via one circadian questionnaire may not be translatable to those of another. Nevertheless, to date, no systematic and hierarchical analysis of the content of the most commonly used circadian self-report questionnaires, including in terms of content overlap, has been carried out.

An original method for comparing item content between questionnaires and quantifying their degree of overlap was developed in the field of affective disorders (Fried, 2017). That study used the Jaccard similarity coefficient (ϱ) to quantify the degree of overlap (i.e., homogeneity) of content extracted from self-reported depression questionnaires (Fried, 2017). Since then, this methodological framework has been applied to several other complaints and disorders (Charvet et al., 2022; Chrobak et al., 2018, 2021; Fried, 2017; Fried et al., 2022; Karstoft & Armour, 2022; Visontay et al., 2019). We recently applied this method to various sleep disorder questionnaires designed for adults (Coelho et al., 2023; Gauld et al., 2023, 2024). However, to the best of our knowledge, the method has not yet been applied to circadian self-report questionnaires for adults.

In this study, our main objective was to better distinguish the different circadian manifestations (actual sleep time, preferred time to sleep, and so forth) and related dimensions that are investigated by commonly used circadian self-report questionnaires for adults, and to analyze and quantify the degree to which these questionnaires overlap in terms of content (i.e., the degree of homogeneity). To this end, we performed content analysis, applied visualization methods, and calculated Jaccard indices. Finally, we make recommendations regarding the circumstances in which some circadian questionnaires could prove more useful than the others.

## Methods

The method and vocabulary used in this study are based on those applied in previous studies that have performed content overlap analyses (Charvet et al., 2022, 2022; Chrobak et al., 2018, 2021; Coelho et al., 2023; Fried, 2017; Fried et al., 2022; Gauld et al., 2023, 2024; Karstoft & Armour, 2022; Visontay et al., 2019).

### Selection of self-report questionnaires

To identify circadian self-report questionnaires for adults, we conducted a systematic search of PubMed using the following search terms: ((Circadian [Title/Abstract] OR Chronotype [Title/Abstract]) AND (Scale [Title/Abstract] OR Questionnaire [Title/Abstract])) AND (Validation [Title/Abstract] OR Psychometric [Title/Abstract]). We focused on articles that developed, translated, validated or reviewed one or more circadian questionnaires. Only articles published in English were included. No limitation on publication date was set. The results of this systematic search were compared with the findings of three previous comprehensive reviews on circadian self-report measures in adults to ensure completeness (Adan et al., 2012; Levandovski et al., 2013; Putilov, 2017). In total, 19 questionnaires were identified.

To evaluate the questionnaires specifically in terms of heterogeneity and overlap, we only included self-reported circadian questionnaires published in peer-reviewed journals that had been validated sufficiently in terms of their psychometric properties (this was not the case, for example, for the Marburger questionnaire) (Reinberg et al., 1981). We excluded questionnaires created to assess general sleep disturbances, rather than circadian rhythms specifically, in adults (e.g., the Pittsburgh Sleep Quality Index) (Buysse et al., 1989). Questionnaires including only items from previous scales that were rewritten (e.g., the Basic Language Morningness Scale) (Brown, 1993), reversed (e.g., the Evening Chronotype Scale) (Kim & Lee, 2021), recontextualized (e.g., the MCTQ for shift-workers) (Juda et al., 2013), or remixed (e.g., the Morningness–Eveningness-Stability Scale improved [MESSi]) (Randler et al., 2016) and marginally used (< 200 citations in Google Scholar by July 2024) were also excluded. Conversely, the CSM (includes items from the MEQ and the DTQ) and the reduced Morningness-Eveningness Questionnaire (rMEQ; includes items from the MEQ) were included due to their wide use (1366 and 848 citations, respectively).

According to the above criteria, 14 self-report circadian questionnaires for adults were selected: the 19-item MEQ (Horne & Ostberg, 1976), the 32-item MCTQ (Roenneberg et al., 2003), the 13-item CSM (Smith et al., 1989), the 5-item rMEQ (Adan & Almirall, 1991), the 7-item Diurnal Type Questionnaire (DTQ) (Torsvall & Akerstedt, 1980), the 20-item Circadian Type Questionnaire (CTQ) (Folkard et al., 1979), the 14-item Chronotype Questionnaire (CQ) (Ogińska, 2011), the 3-item CIRcadian ENergy Scale (CIRENS) (Ottoni et al., 2011), the 18-item Caen Chronotype Questionnaire (CCQ) (Dosseville et al., 2013), the 11-item Circadian Type Inventory (CTI) (Di Milia et al., 2004), the 34-item Circadian Amplitude and Phase Scale (CAPS) (Di Milia et al., 2011), the 15-item Sleep, Circadian Rhythms, And Mood Questionnaire (SCRAM) (Byrne et al., 2017), the 6-item Sleep Regularity Questionnaire (SRQ) (Dzierzewski et al., 2021), and the Single-Item Chronotyping (SIC) tool (Putilov et al., 2021).

### Extraction and selection of items

An item was defined as any question that has to be answered by the respondent, and the number of extracted items corresponded to the number of questions included in each questionnaire. We extracted a total of 161 items from the 14 circadian self-report questionnaires (**Supplementary Materials 1**).

### Extraction of manifestations from items

A “manifestation” is defined as any unit of analysis of content extracted from a questionnaire item. The extraction of manifestations from items involved three steps.

#### Step 1: extraction from items in each questionnaire

We used a double-blind method involving two experts in sleep and circadian medicine (JC and JAMF) to increase the reliability of the manifestation extractions. When disagreement arose between the experts, a consensus was sought by referring to a third expert (JT). The manifestations identified in each questionnaire were grouped as follows.

First, we extracted all units of analysis from the 161 items across the 14 circadian questionnaires. Units of analysis are defined as the smallest, indivisible features within the sentences of the items. For instance, we extracted three units of analysis (i.e., “peak performance,” “mentally exhausting test,” and “feeling best rhythm”) from item 8 of the CSM (“You wish to be at your peak performance for a test which you know is going to be mentally exhausting and lasting for two hours. You are entirely free to plan your day, and considering only your own ‘feeling best’ rhythm, which ONE of the four testing times would you choose?”). Then, manifestations were created by “splitting” or “lumping” the unit of analysis (Gauld et al., 2023). For instance, the “peak performance” and “mentally exhausting tests” were combined into a single “mental activity” manifestation (lumping), while the “mental activity” and “feeling best rhythm” remained as two distinct manifestations (splitting). Manifestations judged as unrelated to circadian rhythms were excluded, and consequently, items assessing only these unrelated manifestations (*n* = 19) were omitted (e.g., CTI items about appropriate sleep duration [“Do you tend to need more sleep than other people?”] and CTI items pertaining to the ability to cope with drowsiness [“If you have something important to do but feel very drowsy, can you overcome your drowsiness?"]). Thus, 40 distinct circadian manifestations from 142 items were finally included.

Second, we differentiated among three kinds of manifestations. “Compound manifestation” refers to a manifestation extracted from an item that evaluates at least two distinct manifestations (e.g., two manifestations were extracted and split from item 8 of the CSM). A “specific manifestation” is derived from an item that measures a single manifestation. For example, the first item of the MEQ concerns only the “free rise time" manifestation. When the same manifestation was extracted from two or more items from a given questionnaire containing both specific and compound manifestations, it was considered specific.

Finally, idiosyncratic manifestation refers to a manifestation measured using an item appearing in only one questionnaire. For example, the manifestation “bedtime during workdays” was extracted only from the first item of the MCTQ.

#### Step 2: harmonization of manifestations

To avoid redundancy and biases in the analyses, we combined manifestations referring to a similar construct across questionnaires. For instance, the second item of the MEQ (“What time would you go to bed if you were entirely free to plan your evening?”) and item 8 of the MCTQ (“During free days, I go to bed at _____ o'clock”) were combined into the “free bedtime" manifestation.

To better align our wording with the literature, we used an original, conservative approach based on previous work involving the extraction and harmonization of manifestations in the ICSD-3 (American Academy of Sleep Medicine, 2014; Gauld et al., 2021), and on the clinical dimensions identified in previous literature (Adan et al., 2012; Levandovski et al., 2013; Putilov, 2017).

#### Step 3: hierarchical classification of manifestations into dimensions

**Fig. 1:**
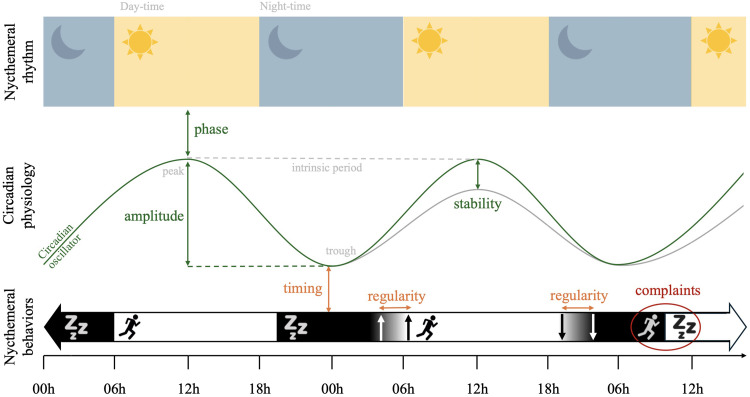
Schematic representation of the five circadian dimensions. **Nycthemeral rhythm**: 24 h period including alternation of daytime and nighttime due to solar exposition. **Circadian physiology (green)**: near-24 h patterns of physiological parameters, including peaks (maximum over the nycthemeral rhythm), troughs (minimum over the nycthemeral rhythm), and periods (delay between two identical points). The **phase** (timing of the peak and trough across the nycthemeral rhythm), **amplitude** (difference between the peak and trough), and **stability** (difference between periods across days). **Nycthemeral behaviors (orange)**: near-24 h patterns of behaviors with sleep (black) and wake (white) alternation. The **timing** (actual sleep–wake behaviors related to physiological circadian rhythms) and **regularity** (difference in behavioral nycthemeral timing across days) are shown. **Circadian complaints (red)**: clinical symptoms of sleep disturbance experienced by an individual depending on their physiological circadian and behavioral nycthemeral rhythms. These dimensions are components of **circadian health**.

Finally, we organized the extracted manifestations into five dimensions, in line with the definitions commonly used in circadian rhythm studies (Adan et al., 2012; Di Milia & Folkard, 2021; Dijk & Duffy, 2020; Roenneberg et al., 2003) and taking into account the dimensions extracted through factorial analysis of the selected questionnaires in validation studies, where available (i.e., for the CSM, DTQ, CTQ, CQ, CCQ, and CTI) (Di Milia et al., 2004; Dosseville et al., 2013; Folkard et al., 1979; Ogińska, 2011; Smith et al., 1989; Torsvall & Akerstedt, 1980). The five circadian dimensions are represented schematically in Fig. 1.

- The “circadian phase” dimension was defined as the timing of the peak and trough of the physiological circadian rhythm across the daytime and nighttime, according to the definition proposed by Di Milia and Folkard (2021). Individuals are categorized as having a morning, neutral, or evening physiological type according to their synchronization with day–night alternation (also called the nycthemeral rhythm) (Di Milia & Folkard, 2021).
- The “circadian amplitude and stability” dimension was defined as the difference between the peak and trough of the physiological circadian rhythm (i.e., the degree of circadian oscillation), and the constancy in an individual's circadian amplitude from one day to the next (i.e., the consistency of the circadian oscillation), according to the definition proposed by Di Milia and Folkard (2021). Concerning amplitude, individuals are categorized as “vigorous” or “languid” on the basis the difference between the preferred and non-preferred circadian timing (small and large difference, respectively). Concerning stability, individuals are categorized as “flexible” or “rigid” depending on whether they are able to adapt their physiological circadian amplitude (Di Milia & Folkard, 2021). These two dimensions were regrouped because of the difficulty of differentiating between items exploring amplitude versus stability, and based on previous data-driven factorial analyses. For instance, the first item of the CTQ (“If you have been out very late at a party, how easy do you find it to ‘sleep in’ the following morning if there is nothing to prevent you doing so?”) was classified into the stability factor (Folkard et al., 1979), while item 8 of the CTI (“If you go to bed very late do you need to sleep in the following morning?”) was classified into the amplitude factor (Di Milia et al., 2004). When extracting manifestations via our content analysis, similar difficulties in differentiating items exploring circadian amplitude and/or stability were encountered. For instance, item 5 of the CTI (“Do you enjoy working at unusual times of day or night?”) can either correspond to a vigorous (i.e., small difference between usual and unusual times of day) or flexible (i.e., ability to deviate from usual times of day) circadian rhythm. Interestingly, the ability to adjust rapidly to shift work (i.e., stability) has been found to be negatively related to the amplitude of the circadian rhythm (Reinberg et al., 1978). Lastly, the “amplitude and stability" dimensions have often been studied together in previous research (Bagheri Hosseinabadi et al., 2019, 2021; Di Milia & Folkard, 2021; Jafari Roodbandi et al., 2015; Zare et al., 2017) and were even combined in a validation study of a circadian rhythm questionnaire that incorporated items from earlier scales (Randler et al., 2016).
- The “nycthemeral timing” dimension was defined as actual sleep–wake behaviors related to the nycthemeral rhythm, according to the approach of Roenneberg et al. (2003). This dimension is used to quantify the agreement between the behaviors of individuals and the nycthemeral rhythm, particularly in relation to external factors (e.g., constraints related to social requirements or light exposure). Individuals are categorized as having a morning, neutral, or evening behavioral timing according to their synchronization with day–night alternation (also called the nycthemeral rhythm). In the absence of constraints, nycthemeral timing is closely linked with circadian phase. But under the influence of constraints, a morning type individual may have evening timing, while an evening type individual may have morning timing.
- The “nycthemeral regularity” dimension was defined as the difference in behavioral timings across days (i.e., the consistency of actual sleep–wake behaviors) according to the systematic review of Fischer et al. (2021). Individuals are categorized as “regular” or “irregular" (i.e., as having a large or small difference in behavioral timings across days, respectively). In the absence of constraints, nycthemeral regularity is closely linked with circadian stability. But under the influence of constraints, a rigid individual may become irregular, while a flexible individual may remain regular.

The “circadian phase” and “circadian amplitude and stability” dimensions pertain to the circadian physiology of an individual (Di Milia & Folkard, 2021), while “nycthemeral timing” and “nycthemeral regularity" pertain to nycthemeral behaviors (American Academy of Sleep Medicine, 2014; Fischer et al., 2021; Roenneberg et al., 2003).

- Finally, the “circadian complaints” dimension was defined as the clinical symptoms of sleep disturbance that may be experienced by an individual depending on their physiological circadian and behavioral nycthemeral rhythms, according to the nosography of the sleep disturbances associated with CSWRDs in the ICSD-3 (American Academy of Sleep Medicine, 2014).

The details of the manifestations extracted from the questionnaire items, including the harmonization and its hierarchical classification, are presented in **Supplementary Materials 1**.

#### Data aggregation

Finally, we coded the extracted circadian manifestations using a three-point scale: a value of 1 was assigned to a questionnaire capturing a specific or idiosyncratic manifestation, with a value of 2 being assigned to one capturing compound manifestations and a value 0 being assigned when a manifestation was not covered by a given questionnaire.

### Statistical analysis

#### Number and distribution of manifestations

We analyzed the number of circadian manifestations extracted from the questionnaires for all five dimensions (“circadian phase”, “circadian amplitude and stability”, “nycthemeral timing”, “nycthemeral regularity”, and “circadian complaints"), and the distribution of the circadian manifestations, and we identified the questionnaires with the highest number of specific and compound manifestations (to assess possible lack of precision due to the inclusion of items constructed on the basis of at least two distinct manifestations).

#### Content overlap analysis

The Jaccard index, widely used to measure content overlap between questionnaires in a binary manner (0, no overlap; 1, complete overlap), is calculated by the following equation:

s/(u1+u2+s)

where *s* represents the number of manifestations shared by two questionnaires, and *u1* and *u2* represent the number of manifestations unique to each questionnaire (Fried, 2017). In line with the standard methodology (Fried, 2017), we used the Jaccard index classification in Straightforward Statistics for the Behavioral Sciences (Evans, 1996): very weak, 0.00–0.19; weak, 0.20–0.39; moderate, 0.40–0.59; strong, 0.60–0.79; very strong, 0.80–1.

We computed the Jaccard index to quantify the content overlap of the 14 questionnaires. We also conducted Spearman correlations between the Jaccard indices and the total number of manifestations captured by each questionnaire, and between the number of specific and compound manifestations. The goal was to investigate whether questionnaire length and the presence of specific or compound manifestations played a role in overlap. In addition, we performed pairwise analyses of the overlap between each questionnaire and the other 13 questionnaires to identify which questionnaires were most similar in terms of content. Finally, we performed a stratified analysis of content overlap by computing the Jaccard index for manifestations in each of the five circadian dimensions, to determine which manifestations contributed most to questionnaire heterogeneity.

#### Visualization of content overlap

We plotted the distribution of the manifestations measured by each questionnaire using the Python package *plotly*.^1^ The interactive radar plot in Fig. 4 shows the different manifestations covered by each questionnaire. The manifestations were allocated to angles while the questionnaires were assigned to radii. The sunburst plot in Fig. 5 shows the hierarchical arrangement of the manifestations within the proposed dimensions. These interactive figures are available online and allow clinicians and researchers to easily visualize the different manifestations evaluated by the questionnaires.

#### Availability and reproducibility of the results

Based on previous sleep overlap studies (Coelho et al., 2023; Gauld et al., 2023, 2024), and to promote openness and sharing, all of our data, analysis results, figures, tables, and code are available in a GitHub repository.^2^ The repository contains the analysis files in html format.^3^ To aid reproducibility, we also set up a Binder repository^4^ allowing our results to be fully reproduced online. Finally, we adapted our analysis files so that the same metrics calculated in this study can be emulated, along with the same figures and tables, if the formatting specified in the dedicated GitHub repository is applied.^5^

## Results

### Number and distribution of manifestations

The 14 questionnaires analyzed in this study included 161 items, from which we extracted a total of 197 circadian manifestations via splitting or lumping (Table 1). After harmonization, we identified a total of 40 distinct circadian manifestations from 142 items, classified into the five circadian dimensions.

**Table 1:** Average Jaccard index number of items, and number of specific and compound manifestations in the 14 circadian questionnaires.

	Average Jaccard Index	Number of items	Specific manifestations	Compound manifestations	Total number of extracted manifestations	Total number of distinct manifestations
**CAPS**	0.152	24	18	12	30	16
**CTQ**	0.167	16	11	10	21	14
**CSM**	0.255	13	9	8	17	12
**MEQ**	0.251	19	14	10	24	12
**MCTQ**	0.077	24	24	0	24	11
**CQ**	0.222	14	10	8	18	13
**CCQ**	0.177	16	15	2	17	10
**CTI**	0.143	10	6	8	14	8
**DTQ**	0.192	7	4	6	10	8
**SCRAM**	0.150	5	4	2	6	6
**rMEQ**	0.202	5	4	2	6	5
**SRQ**	0.009	4	4	0	4	4
**CIRENS**	0.052	3	3	0	3	1
**SIC**	0.052	1	0	3	3	3

	**0.150** (mean)	**161** (total)	**126** (total)	**71** (total)	**197** (total)	**123** (total)

CAPS: Circadian Amplitude Phase Scale; CTQ: Circadian Type Questionnaire; CSM: Composite Scale Morningness; MEQ: Morningness Eveningness Questionnaire; MCTQ: Munich ChronoType Questionnaire; CQ: Chronotype Questionnaire; CCQ: Caen Chronotype Questionnaire; CTI: Circadian Type Inventory; DTQ: Diurnal Type Questionnaire; SCRAM: Sleep, Circadian Rhythms, And Mood; rMEQ: reduced Morningness Eveningness Questionnaire; SRQ: Sleep Regularity Questionnaire; CIRENS: CIRcadian ENergy Scale; SIC: Single-Item Chronotyping.

**Fig. 2:**
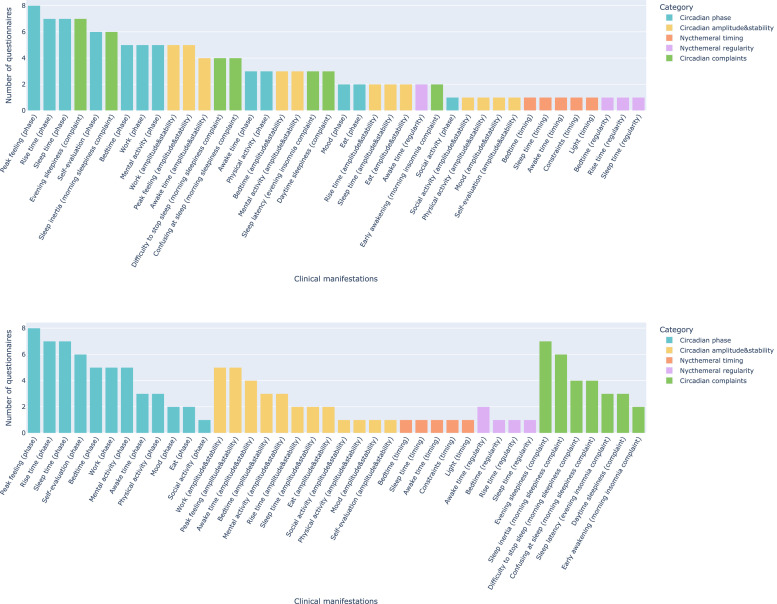
Manifestations covered by the 14 circadian questionnaires. **(Top)** Plot showing the most and least frequent dimensions. **(Bottom)** Plot showing the most and least frequent dimensions among the five dimensions identified previously (American Academy of Sleep Medicine, 2014; Gauld et al., 2021).

Fig. 2 shows the frequencies of the circadian manifestations, ordered from most to least frequent for all circadian dimensions. Regarding the five dimensions, “circadian phase” was the most common, appearing in 12 of the 14 questionnaires. “Peak feeling (phase)” was the most common manifestation within the “circadian phase” dimension (appearing in 8 out of 14 questionnaires; 57.0 %), “evening sleepiness (complaints)” was the most common manifestations in the “circadian complaints” dimension (appearing in 7 out of 14 questionnaires; 50.0 %), “peak feeling (amplitude and stability)” and “work (amplitude and stability)” were the most common manifestations in the “circadian amplitude and stability” dimension (appearing in 5 out of 14 questionnaires; 35.7 %), and “awake time (regularity)” was the most common manifestation in the “nycthemeral regularity" dimension (appearing in 2 out of 14 questionnaires; 14.3 %).

**Fig. 3:**
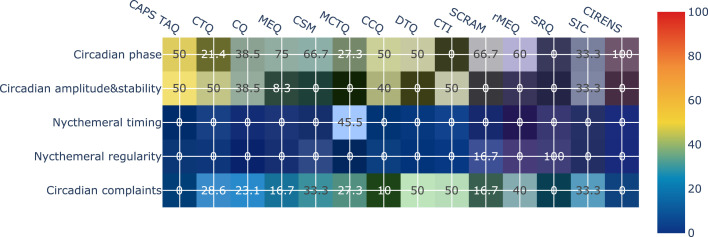
Distribution of manifestations in each questionnaire across the five dimensions. CAPS, Circadian Amplitude Phase Scale; CTQ, Circadian Type Questionnaire; CSM, Composite Scale Morningness; MEQ, Morningness-Eveningness Questionnaire; MCTQ, Munich ChronoType Questionnaire; CQ, Chronotype Questionnaire; CCQ, Caen Chronotype Questionnaire; CTI, Circadian Type Inventory; DTQ, Diurnal Type Questionnaire; SCRAM, Sleep, Circadian Rhythms, And Mood Questionnaire; rMEQ, reduced Morningness-Eveningness Questionnaire; SRQ, Sleep Regularity Questionnaire; CIRENS, CIRcadian ENergy Scale; SIC, Single-Item Chronotyping.

Fig. 3 shows the distribution of the manifestations in each questionnaire across the five dimensions. Two questionnaires contained no manifestations in the “circadian phase” dimension (SRQ and CTI) (Di Milia et al., 2004; Dzierzewski et al., 2021), and three questionnaires contained no manifestations in the “circadian complaints” dimension (CAPS, SRQ, and CIRENS) (Di Milia et al., 2011; Dzierzewski et al., 2021; Ottoni et al., 2011). The “circadian amplitude and stability” dimension appeared in seven questionnaires (CAPS, CQ, MEQ, CTQ, CCQ, CTI, and SIC) (Di Milia et al., 2004, 2011; Dosseville et al., 2013; Folkard et al., 1979; Horne & Ostberg, 1976; Ogińska, 2011), the “nycthemeral regularity” dimension appeared in two questionnaires (SCRAM and SRQ) (Byrne et al., 2017; Dzierzewski et al., 2021), and the “nycthemeral timing" dimension appeared in only one questionnaire (MCTQ) (Roenneberg et al., 2003). The number and distribution of manifestations across dimensions are available in our online analysis notebook.^6^

The questionnaires containing the largest number of distinct circadian manifestations were the CAPS (n = 16/40) and the CTQ (*n* = 14/40) (Table 1). Among the 197 manifestations extracted, 71 were compound (36.0 %), and 126 were specific (64.0 %). Among the 40 distinct manifestations, 13 were idiosyncratic (32.5 %), appearing only in one questionnaire. The questionnaire with the largest number of compound manifestations was the CAPS (*n* = 12), followed by the CTQ (*n* = 10) and the MEQ (*n* = 10). The MCTQ, SRQ, and CIRENS contained only specific manifestations (Table 1).

### Content overlap analysis

Table 1 presents the average Jaccard index, number of items, and number of specific and compound manifestations for each questionnaire. The mean Jaccard index (overlap) between the questionnaires was 0.150 ± 0.077), indicating very low similarity. The questionnaires with the highest average Jaccard index were the CSM (0.255) and MEQ (0.251), followed by the CQ (0.222), and rMEQ (0.202). These four questionnaires explored both the “circadian phase” and “circadian complaints" dimensions.

**Fig. 4:**
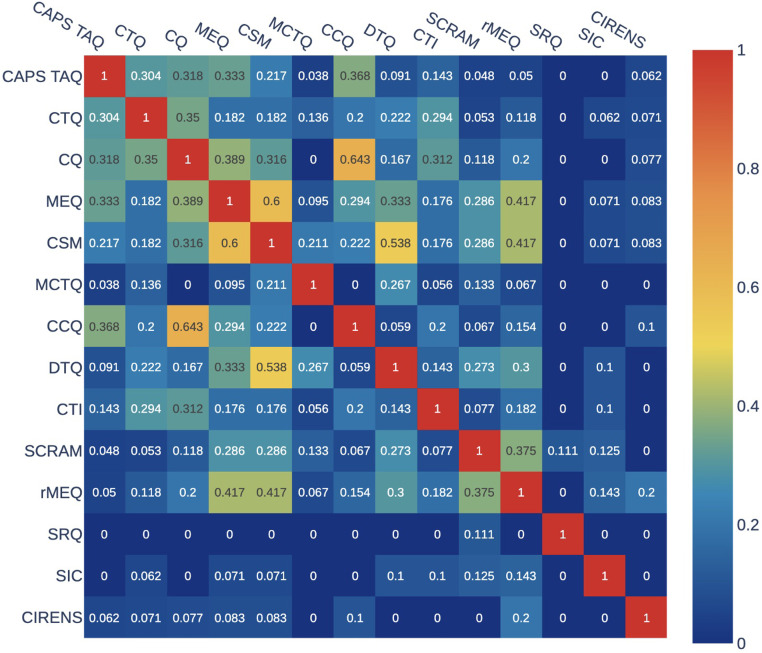
Jaccard index denoting the overlap in item content in pairwise comparisons of the 14 circadian questionnaires. CAPS, Circadian Amplitude Phase Scale; CTQ, Circadian Type Questionnaire; CSM, Composite Scale Morningness; MEQ, Morningness-Eveningness Questionnaire; MCTQ, Munich ChronoType Questionnaire; CQ, Chronotype Questionnaire; CCQ, Caen Chronotype Questionnaire; CTI, Circadian Type Inventory; DTQ, Diurnal Type Questionnaire; SCRAM, Sleep, Circadian Rhythms, And Mood Questionnaire; rMEQ, reduced Morningness-Eveningness Questionnaire; SRQ, Sleep Regularity Questionnaire; CIRENS, CIRcadian ENergy Scale; SIC, Single-Item Chronotyping.

None of the 14 questionnaires explored more than three dimensions. Moreover, the SRQ only explored nycthemeral regularity, and the CIRENS only explored the “circadian phase” dimension. The correlations of the Jaccard index with the total number of extracted circadian manifestations and the total number of compound circadian manifestations were significant (ϱ = 0.579, *p* = 0.003 and ϱ = 0.570, *p* = 0.033, respectively), but the correlation between the Jaccard index and the total number of specific circadian manifestations was not (ϱ = 0.349, *p* = 0.222). Regarding the pairwise analysis of overlap, the CCQ and CQ had the highest pairwise overlap value (0.643), followed by the CSM and MEQ (0.600) and the CSM and DTQ (0.538). Conversely, the MCTQ did not overlap at all with the CQ or the CCQ. The SIC tool and the CIRENS had a very small amount of pairwise overlap with a few questionnaires. The SRQ did not overlap at all with 12 of the 13 other questionnaires (Fig. 4). Finally, the results of the content overlap analysis stratified by dimension are reported in Table 2. The overlap among the 14 questionnaires was highest for the “circadian complaints” dimension (0.257), followed by “circadian phase” (0.254), “circadian amplitude and stability” (0.223), and “nycthemeral regularity” (0.250). The manifestations in the “nycthemeral timing" dimension were idiosyncratic (only appearing in the MCTQ) and thus had no overlap.

**Table 2:** Manifestations in each dimension, and overlap (average Jaccard index) between dimensions.

	Average Jaccard Index	Included manifestations	Number of manifestations
**Circadian phase**	0.254	**Preference for**: morning time “Awake time” and “Rise time", daytime “Social activity”, “Work”, “Mental activity”, “Physical activity”, “Peak feeling”, “Mood”, and “Eat", evening time “Bedtime” and “Sleep time" circadian preference “Self-evaluation”.	12
**Circadian amplitude & stability**	0.223	**Strength of the circadian preference** thought different times-of-day and items exploring the **adaptability to usual or unusual**: morning time “Awake time” and “Rise time", daytime “Social activity”, “Work”, “Mental activity”, “Physical activity”, “Peak feeling”, “Mood”, and “Eat", evening time “Bedtime” and “Sleep time" circadian amplitude & stability “Self-evaluation”	12
**Nycthemeral timing**	0.0	**Behaviors:** morning time “Awake time” evening time “Bedtime” and “Sleep time" external factors “Constraints” and “Light".	5
**Nycthemeral regularity**	0.250	**Variability of:** morning time “Awake time” and “Rise time" evening time “Bedtime” and “Sleep time"	4
**Circadian complaints**	0.257	**Symptoms experienced** during morning time “Sleep inertia”, “Difficulty to stop sleep”, “Confusing at sleep”, and “Early awakening", daytime “Daytime sleepiness” evening time “Sleep latency” and “Evening sleepiness".	7

	0.197		40

### Visualization of content overlap

**Fig. 5:**
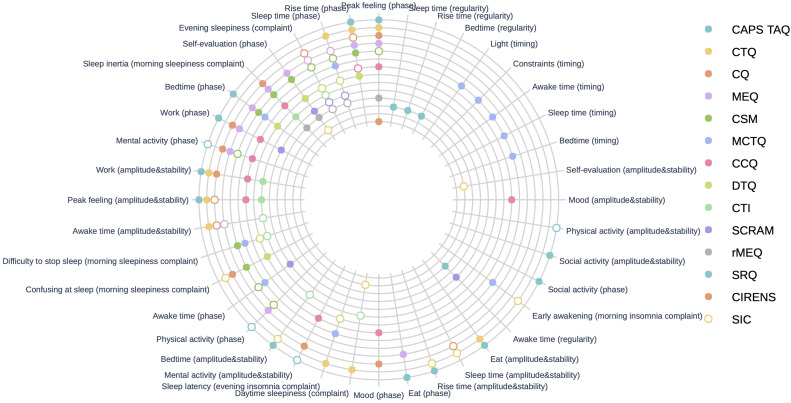
Overlap of manifestations in the 14 selected circadian questionnaires. Colored circles indicate specific manifestation, and empty circles indicate compound manifestations. See also the interactive online version of this figure (https://chart-studio.plotly.com/~vincent.martin/97.embed). CAPS, Circadian Amplitude Phase Scale; CTQ, Circadian Type Questionnaire; CSM, Composite Scale Morningness; MEQ, Morningness-Eveningness Questionnaire; MCTQ, Munich ChronoType Questionnaire; CQ, Chronotype Questionnaire; CCQ, Caen Chronotype Questionnaire; CTI, Circadian Type Inventory; DTQ, Diurnal Type Questionnaire; SCRAM, Sleep, Circadian Rhythms, And Mood Questionnaire; rMEQ, reduced Morningness-Eveningness Questionnaire; SRQ, Sleep Regularity Questionnaire; CIRENS, CIRcadian ENergy Scale; SIC, Single-Item Chronotyping.

**Fig. 6:**
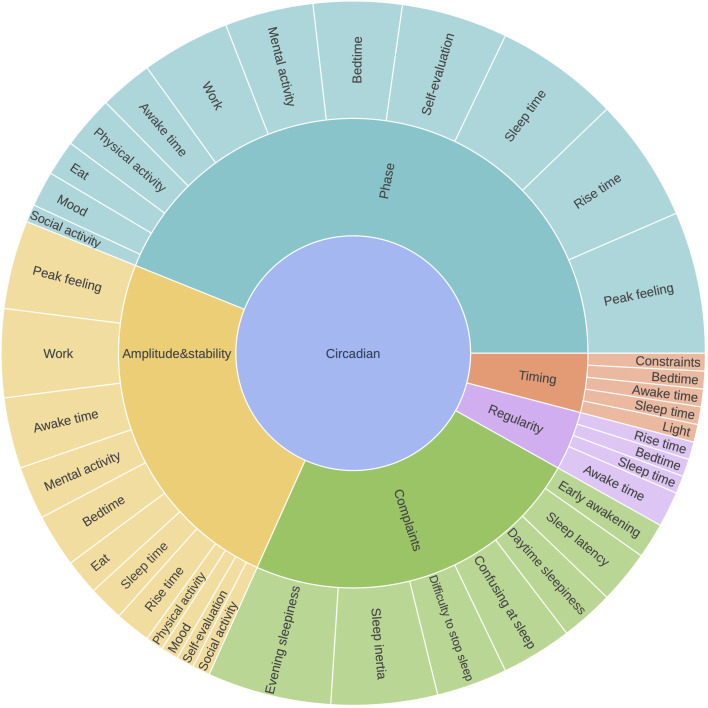
Sunburst plot showing the hierarchical arrangement of manifestations according to the five dimensions, weighted by the number of occurrences. Red, nycthemeral timing; yellow, circadian amplitude and stability, purple: circadian complaints; light blue, circadian phase; dark blue, nycthemeral regularity. Please also see the interactive online version of this figure (https://chart-studio.plotly.com/~vincent.martin/95.embed).

Fig. 5 shows the content overlap of the 14 questionnaires (see https://chart-studio.plotly.com/~vincent.martin/97.embed for an interactive online version). This figure clearly shows the manifestations evaluated by each questionnaire. The hierarchical arrangement of the manifestations according to dimension is shown in the sunburst plot in Fig. 6 (see https://chart-studio.plotly.com/~vincent.martin/95.embed for an interactive online version).

## Discussion

### Key findings

This study is the first systematic, quantitative analysis of content overlap among 14 self-report questionnaires commonly used to assess circadian rhythms in adults. Our results highlight the heterogeneity in content among the questionnaires (mean ϱ = 0.150) and demonstrate the different types of circadian manifestations investigated by each questionnaire. Although the psychometric properties of all of the questionnaires assessed in this study have been validated (Adan et al., 2012; Di Milia et al., 2013; Levandovski et al., 2013), the differences found in their content suggest that they should not be used interchangeably. These findings make it possible to refine the use of these questionnaires which complement each other for a more exhaustive and appropriate characterization of circadian rhythms.

### Circadian physiology

Manifestations in the “circadian phase” dimension were the most prevalent (*n* = 12) across the 14 questionnaires. Furthermore, they accounted for 16 of the 24 manifestations covered by the MEQ, which was the first circadian questionnaire to be published (Horne & Ostberg, 1976). The MEQ was created following the first description of circadian rhythms in humans, by Aschoff, (1965). Experimental protocols assessed the “ intrinsic period” of circadian rhythms (i.e., the delay between two identical points in the cycle), which ultimately determines the synchronization with the day/night cycle and by extension, the degree of preference for morningness (period < 24 h) or eveningness (period > 24 h) (Czeisler et al., 1999). The “circadian phase” dimension exhibited weak overlap with the other dimensions (ϱ = 0.254). Most instruments focus on both the preferred bedtime/rise time and the preferred time to carry out seven daily activities (e.g., the MEQ) (Horne & Ostberg, 1976). However, other instruments refer more to the preferred sleep time and wake time (e.g., the SCRAM) (Byrne et al., 2017) or self-assessment of circadian preference (e.g., the SIC tool) (Putilov et al., 2021). In this study, the “peak feeling (phase)” manifestation was the most prevalent manifestation in the “circadian phase” dimension (8/12 questionnaires). Notably, the CTI (Di Milia et al., 2004) and SRQ (Dzierzewski et al., 2021) do not assess the “circadian phase" dimension, and these questionnaires should thus be used to assess other circadian phenomena.

The CTQ was the second circadian questionnaire to be published (Folkard et al., 1979). We found that its main dimension is “circadian amplitude and stability” (12/21 manifestations), where these features of circadian physiology are better predictors of adaptation to shift work than the “circadian phase” (Aschoff, 1978). Although we classified amplitude- and stability-related manifestations together, this dimension still exhibited the smallest degree of overlap with the others (ϱ = 0.223). Questionnaires variously rely on the evaluation of the amplitude/stability of sleep and wake times, bed, and rise times, or the preferred timing of seven daily activities. In our analysis, “peak feeling (amplitude and stability)” and “work (amplitude and stability)” were the most prevalent circadian manifestations of the circadian “amplitude and stability" dimension (5/7 questionnaires). As described above, amplitude and stability are difficult to distinguish in terms of content but also in factorial analyses (Di Milia et al., 2004; Folkard et al., 1979). Interestingly, the ability to adjust rapidly to shift work (i.e., stability) has been found to be negatively related to the amplitude of the circadian rhythm (Reinberg et al., 1978), which suggests that our hierarchical classification has clinical significance. Despite their importance for adaptation to schedules, the amplitude and stability dimensions are not covered by the DTQ, CSM, or rMEQ (Adan & Almirall, 1991; Smith et al., 1989; Torsvall & Akerstedt, 1980). Today, these questionnaires are largely neglected compared to the MEQ (7470 citations in Google Scholar by July 2024), MCTQ (2843) and CSM (1366). However, it is important to note that, from a physiological perspective, amplitude and stability represent distinct dimensions. This distinction is evidenced by differences observed with aging: amplitude tends to decrease, whereas stability tends to increase (Di Milia & Folkard, 2021; Hood & Amir, 2017). Since these are important clinical dimensions, particularly for adapting to shift work, further studies should aim to better characterize and differentiate them by dedicated reliable and valid questionnaire. Objective measures of circadian rhythms, such as salivary or plasma melatonin, urinary 6-sulfatoxymelatonin, or core body temperature, could be instrumental in achieving this goal (Reid, 2019). This is particularly important for circadian stability, as there is currently no standardized and validated physiological biomarker which underscores the need for further research into the physiological underpinnings of this dimension, initially described in a clinical application (Folkard et al., 1979). These efforts would allow for clearer differentiation and systematic integration into physiological and clinical studies of circadian rhythms.

### Nycthemeral behaviors

Roenneberg et al. (2003) introduced the MCTQ, a questionnaire used to identify behavioral markers for the circadian phase (i.e., the mid-sleep point during free days) rather than theoretical circadian preference. Interestingly, this questionnaire showed very weak overlap with the others (ϱ = 0.077) in our analysis. The MCTQ allows for evaluation of the overall physiology of an individual (i.e., the “circadian phase” dimension) based on the consequences of social and environmental constraints for sleep–wake behaviors (i.e., the nycthemeral timing dimension specific to this questionnaire). Only the MCTQ allows self-report assessment of the circadian phase and its alignment with sleep–wake behaviors. This approach is in line with the clinical diagnostic criteria for CSWRDs (American Academy of Sleep Medicine, 2014), defined as “sleep disturbance resulting from […] misalignment between the endogenous circadian rhythm and exogenous factors." A morning shift might be convenient for an individual with a morning phase but may cause misalignment between the endogenous rhythm (i.e., phase) and exogenous rhythm (i.e., timing) for an individual with an evening phase.

In 2017, “nycthemeral regularity” appeared for the first time in a circadian questionnaire, namely, the SCRAM (Byrne et al., 2017), before being the subject of a self-report questionnaire in 2021 (the SRQ; (Dzierzewski et al., 2021). “Nycthemeral regularity” completes the assessment of global alignment between the “circadian phase” and “nycthemeral timing" by measuring day-to-day circadian disruptions caused by irregularities in sleep–wake behaviors (Vetter, 2020). This day-to-day irregularity is mainly a consequence of the constraints imposed by shift work (Wickwire et al., 2017). Individuals with a high circadian amplitude (i.e., languid) and/or high circadian stability (i.e., rigid) under such conditions are prone to develop a shift work sleep disorder (SWSD). Conversely, individuals with a low circadian amplitude (i.e., vigorous) and/or low circadian stability (i.e., flexibility) are better able to cope with and tolerate shift schedules (Flo et al., 2012). Day-to-day irregularity can also be spontaneous, as suggested by studies that have reported a high prevalence of sleep irregularity among youths and university students (Coelho et al., 2024; Roenneberg et al., 2019). These populations trend to be more vigorous/flexible than older adults and live with fewer constraints, which makes day-to-day variation more likely (Vitiello et al., 1986). Importantly, the SCRAM and the SRQ are the first measures of nycthemeral regularity that do not require prospective completion of a sleep diary or actimetry (Fischer et al., 2021). As evidence accumulates regarding the importance of nycthemeral regularity for sleep and health (Chaput et al., 2020; Sletten et al., 2023; Taillard et al., 2021; Windred et al., 2024), these two questionnaires will become increasingly necessary. Interestingly, evaluations of nycthemeral regularity only consider the regularity of sleep–wake behavior. However, the regularity of daily activities, including meals, may also be important for sleep and health (Deibel et al., 2022). Future questionnaires should consider using items from recently validated scales for the evaluation of meal timing (Chakradeo et al., 2022).

### Circadian health

Manifestations in the “circadian complaints” dimension have been covered since the first circadian questionnaire was published (i.e., the MEQ, in 1976), and we found that they were included in 11 of the 14 questionnaires. This dimension exhibited the largest (albeit still minor) degree of overlap with the other circadian dimensions (ϱ = 0.257).

**Fig. 7:**
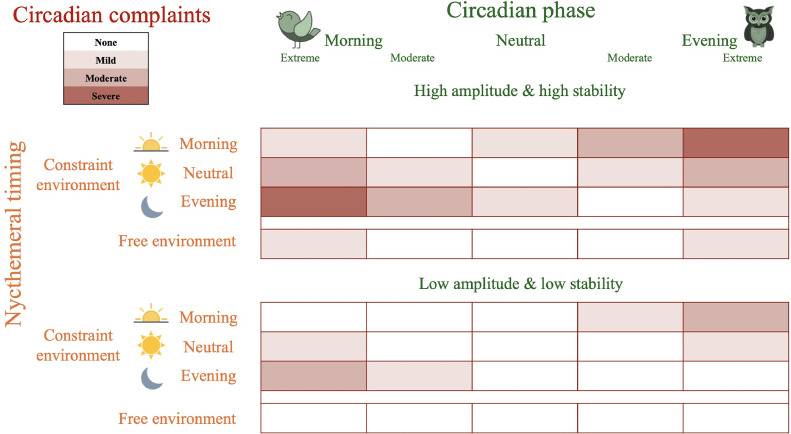
A proposed summary of interactions between different circadian dimensions that may promote circadian health. Circadian physiology (green), encompassing circadian phase (extreme morning, moderate morning, neutral, moderate evening, extreme evening) and circadian amplitude and stability (upper, high amplitude & high stability; lower, low amplitude & low stability). Nycthemeral behaviors (orange), encompassing nycthemeral timing (morning constraint, neutral constraint, evening constraint, free environment). Circadian complaints (red: no, mild, moderate, or severe complaints). Nycthemeral regularity: day-to-day irregularity without advanced or delayed phase.

These circadian complaints are generally not fully explained by a single dimension of circadian physiology (i.e., phase, amplitude and stability) or nycthemeral behaviors (i.e., timing and regularity), but rather are related to a “circadian disruption” between these different dimensions. These circadian disruptions and their clinical consequences are described in the ICSD-3 as CSWRDs (American Academy of Sleep Medicine, 2014). Two forms are particularly important: the circadian misalignment (or circadian desynchronization), i.e., a mismatch between circadian physiology and nycthemeral behaviors due to external constraints (e.g., “advanced sleep-wake phase syndrome”, “delayed sleep-wake phase syndrome”, “shift work sleep syndrome”, “jetlag syndrome”) and the circadian alteration, i.e., a dysfunction of the biological clock *per se* (e.g., “non-24-hour sleep-wake rhythm disorder”, “irregular sleep-wake rhythm disorder"). Fig. 7**.** presents a proposed summary of the interactions between various dimensions of circadian rhythms, which, when disrupted, can lead to circadian complaints of varying severity.

By evaluating both the “nycthemeral timing” and the “circadian phase", the MCTQ allows the measure of the social jetlag (i.e., the difference between the mid-sleep point on freedays and the mid-sleep point en work days), a measure of the severity of the circadian misalignment (Roenneberg et al., 2019; Vetter, 2020). Thus, an individual with a significant social jetlag will have probably, but not necessarily, complaints or clinically significant suffering or disability that are needed to define CSWRDs (American Academy of Sleep Medicine, 2014). Despite this difference, these concepts are frequently associated in clinical practice (the probability of CSWRDs increases with the severity of social jetlag) but rarely in the scientific literature. Better definition and characterization of these two entities is crucial for understanding circadian disturbances in future studies.

It is important to note that CSWRDs due to circadian misalignment do not exist in the absence of external constraints. The reversibility observed during rest days and holidays further supports their presence (American Academy of Sleep Medicine, 2014; Taillard & Mullens, 2018). Furthermore, the hypothesis of a "mismatch between endogenous circadian rhythms and desired sleep-wake schedules" suggests that any individual, even with normal circadian physiology (neutral phase, average amplitude, and stability), may develop a CSWRD when exposed to an extreme sleep-wake schedule. In this regard, only irregular sleep-wake rhythm disorder and non-24-hour sleep-wake rhythm disorder would meet the definition of sleep disorders under the concept of harmful dysfunction (American Academy of Sleep Medicine, 2014; Guichard et al., 2021). This definition refers to a disruption of physiological function (in this case, biological clock) associated with harm (i.e., circadian complaints and related distress or disability) (Gauld et al., 2025; Wakefield, 1992). CSWRDs thus represent a condition at the intersection of an individual's sleep-wake behaviors, physiology, and environment. Their classification as a disorder, rather than a dimension of health, requires further exploration.

Importantly, the six circadian dimensions and their disruption are associated with several physical and mental health outcomes (Chaput et al., 2020; Di Milia et al., 2024; Kobayashi Frisk et al., 2024; Sletten et al., 2023; Steele et al., 2021; Taillard et al., 2021; Zou et al., 2022). As a result, the concept of “circadian health” is beginning to appear in the scientific literature (Fishbein et al., 2021; Gubin et al., 2025; LaBuzetta et al., 2022). *Roenneberg* et al. define it as “*the condition in which circadian clocks are allowed to stably entrain to zeitgebers and thereby establish an appropriate, stable phase relationship to its cyclic environment*” (Roenneberg et al., 2022). In line with the definition of sleep health (Buysse, 2014), we propose to complete this definition as follows: “circadian health is a multidimensional pattern of circadian rhythms, adapted according to individual, social, and environmental demands, that promotes the body's overall physiology and physical and mental well-being. Good circadian health is characterized by agreement between circadian physiology (phase, amplitude, stability) and nycthemeral behaviors (timing, regularity), promoting homeostasis and synchronization of the organism with its environment and leading to an absence of circadian complaints." Our definition complements that of *Roenneberg* et al. because: (i) it takes into account not only the alignment between the endogenous circadian phase and nycthemeral timing, but also circadian amplitude and stability, and nycthemeral regularity, (ii) it emphasizes the impact of circadian rhythms on the body's overall physiology and physical and mental well-being, without limiting itself to the absence of CSWRD. As such, this definition proposes a positive, multidimensional approach to circadian rhythms that can benefit the entire population, both individually and collectively. Importantly, sensitivity and exposure to external synchronizers (e.g., light exposure), which are at the heart of circadian rhythms (Chellappa, 2021), are not covered by any of the circadian questionnaires, except for two items in the MCTQ on light exposition, but they are embedded in the circadian physiology (i.e., sensitivity) and nycthemeral behaviors (i.e., exposure) dimensions.

### Recommendations for the use of circadian questionnaires

None of the 14 circadian questionnaires evaluate all five circadian dimensions. The CSM and MEQ exhibit the highest degree of overlap with the other questionnaires (ϱ = 0.255 and 0.251, respectively) and are the most appropriate questionnaires for specific assessment of the “circadian phase”. The MEQ has the additional advantage of providing validated thresholds for categorizing patients and detecting extreme values ​​in clinical practice; thus, it is recommended for the evaluation and management of CSWRDs (American Academy of Sleep Medicine, 2014; Morgenthaler et al., 2007). The MCTQ is the only questionnaire that evaluates both the “circadian phase” and “nycthemeral timing”, such that it can be used to assess circadian misalignment; this is essential to measure the circadian misalignment in clinical and research settings. Although no MCTQ items directly evaluate nycthemeral regularity, an essential behavioral dimension for complete evaluation of circadian disruption, its ability to quantify circadian misalignment allows for partial assessment of nycthemeral regularity across workdays and free days. For a complete assessment of day-to-day nycthemeral regularity, the SRQ is appropriate, while the SCRAM jointly assesses the “circadian phase” and “circadian complaints". However, the MCTQ does not evaluate circadian amplitude and stability, which is important for adaptation to misalignment. The CTI specifically evaluates this dimension across 10 items, while the CAPS, CTQ, CQ, and CCQ allow for an exhaustive physiological assessment (i.e., of phase and amplitude and stability). The SIC tool uses only one item for such assessment. The DTQ, rMEQ, and CIRENS are brief questionnaires (seven, five, and three items, respectively) that can be used for specific evaluation of the circadian phase. Based on the interactions between the different circadian dimensions, and with a view to providing a pragmatic tool for clinicians and researchers, Table 3 summarizes recommendations regarding the best circadian questionnaire to use depending on the circadian dimensions to be assessed.

**Table 3:** Summary of the recommendations regarding the best circadian questionnaire to use according to the circadian dimensions to be assessed.

Settings	Assessment of circadian phase	Assessment of circadian amplitude & stability	Assessment of circadian phase and amplitude & stability	Assessment of nycthemeral timing	Assessment of nycthemeral regularity
Clinical	MEQ (19)	-	-	MCTQ (24)	
Research	CSM (13)	CTI (10)	CAPS TAQ (24) CTQ (16) CQ (14) CCQ (16)	MCTQ (24)	
Brief	DTQ (7) rMEQ (5) CIRENS (3)	-	SIC (1)	-	SCRAM (5) SRQ (4)

### Limitations

The results and suggestions of this study should be considered in light of certain limitations. First, regarding the selection of questionnaires, several questionnaires that assess circadian rhythms but use rewritten (e.g., the Basic Language Morningness scale), reversed (e.g., the Evening Chronotype Scale), recontextualized (e.g., the MCTQ), or remixed items (e.g., the MESSi) were excluded. This decision was made to avoid overestimating the overlap between questionnaires containing common items but may have led to underrepresentation of certain circadian manifestations/dimensions. Conversely, the inclusion of the CSM and the rMEQ was important because of the widespread use of these questionnaires, but the small degree of overlap between the circadian questionnaires may have been overestimated.

Second, regarding the extraction of items, several items evaluating manifestations not related specifically to circadian rhythms were also excluded, such as sleep duration (in the CTI) and time awareness (in the CAPS). Several studies have reported close interactions between homeostatic and circadian regulation (Van Dongen & Dinges, 2003), and a link between time awareness and circadian amplitude has been hypothesized (Folkard et al., 2006). We chose to focus on circadian manifestations but other sleep manifestations should also be considered in future studies.

Third, the processes used to extract the manifestations (i.e., splitting, lumping, and rewording) may have impacted our content and overlap analysis. For instance, the assumed preference for certain sleep and wake times (e.g., by the MEQ; (Horne & Ostberg, 1976) informed the analysis of sleep and wake times on free days (e.g., using the MCTQ; (Roenneberg et al., 2003), although these two instruments constitute different methods for assessing the circadian phase, each with unique advantages (Di Milia et al., 2013). Although the already minimal overlap between the manifestations in the “circadian phase” dimension may have been overestimated, our double-blind extraction methodology nevertheless ensures good replicability. Further factorial or network analyses could help validate its suitability for use in various populations (Gauld et al., 2020).

## Conclusion

By systematically evaluating the items of 14 commonly used circadian self-report questionnaires for adults, we were able to identify several factors that can inform appropriate questionnaires selection based on the circadian dimensions to be assessed for clinical or research use. Moreover, the present findings may lead to the development of new circadian questionnaires (or refinement of existing ones), with a special mention to the evaluation of nycthemeral timing, which is essential for a diagnosis of circadian misalignment but is largely overlooked by the questionnaires analyzed herein. Furthermore, circadian amplitude and stability, nycthemeral regularity, and other dimensions of sleep (e.g., homeostasis, synchronizers) should be given more consideration. Overall, our content analysis will aid the definition and investigation of circadian health, which stands at the crossroads of physiology and behavior and contributes to good physical and mental health.

## Author statement

All authors have read and agreed to the published version of the manuscript.

Julien Coelho: conceptualization, methodology, validation, formal analysis, writing—original draft preparation, writing—review and editing.

Vincent P. Martin: conceptualization, methodology, software, formal analysis, writing—original draft preparation, writing—review and editing.

Christophe Gauld: conceptualization, methodology, software, formal analysis, writing—original draft preparation, writing—review and editing.

Emmanuel d'Incau: software, validation, writing—review and editing,

Pierre-Alexis Geoffroy: validation, writing—review and editing.

Patrice Bourgin: validation, writing—review and editing.

Pierre Philip: validation, writing—review and editing.

Jacques Taillard: validation, writing—original draft preparation, writing—review and editing.

Jean-Arthur Micoulaud-Franchi: conceptualization, methodology, validation, formal analysis, writing—original draft preparation, writing—review and editing, supervision.

## Funding

This research did not receive any specific grant from funding agencies in the public, commercial, or not-for-profit sectors.

## Declaration of competing interest

The authors declare that they have no known competing financial interests or personal relationships that could have appeared to influence the work reported in this paper.

## Appendix Supplementary materials

Image, application 1

## Footnotes

1 https://github.com/plotly/plotly.py

2 https://github.com/vincentpmartin/circadian.content.analysis

3 https://raw.githack.com/vincentpmartin/circadian.content.analysis/HEAD/jupyter_notebook_circadian_content_analysis.html

4 https://mybinder.org/v2/gh/vincentpmartin/circadian.content.analysis/HEAD?labpath=jupyter_notebook_circadian_content_analysis.ipynb

5 https://github.com/vincentpmartin/generic.content.analysis

6 https://raw.githack.com/vincentpmartin/circadian.content.analysis/HEAD/jupyter_notebook_circadian_content_analysis.html

## References

[bib0001] Adan A., Almirall H. (1991). Horne & Östberg morningness-eveningness questionnaire: A reduced scale. Personality and Individual Differences.

[bib0002] Adan A., Archer S.N., Hidalgo M.P., Di Milia L., Natale V., Randler C. (2012). Circadian typology: A comprehensive review. Chronobiology International.

[bib0003] American Academy of Sleep Medicine, (2014). International classification of sleep disorders, (3rd Edition). Darien, Ill. 10.5664/jcsm.4050 PMC4153104 25221449

[bib0004] Aschoff J. (1978). Features of circadian rhythms relevant for the design of shift schedules. Ergonomics.

[bib0005] Aschoff J. (1965). Circadian rhythms in man. Science (New York, N.Y.).

[bib0006] Bagheri Hosseinabadi M., Ebrahimi M.H., Khanjani N., Biganeh J., Mohammadi S., Abdolahfard M. (2019). The effects of amplitude and stability of circadian rhythm and occupational stress on burnout syndrome and job dissatisfaction among irregular shift working nurses. Journal of Clinical Nursing.

[bib0007] Bagheri Hosseinabadi M., Khanjani N., Biganeh J., Ebrahimi M.H., Pourhashemi E., Roudi E., Avarseji A. (2021). The role of circadian rhythm stability and amplitude in musculoskeletal disorder prevalence and work–family conflict. Nursing Open.

[bib0008] Bauducco S., Richardson C., Gradisar M. (2020). Chronotype, circadian rhythms and mood. Current Opinion in Psychology.

[bib0009] Brown F.M. (1993). Psychometric equivalence of an improved Basic Language Morningness (BALM) scale using industrial population within comparisons. Ergonomics.

[bib0010] Burgess H.J., Eastman C.I. (2008). Human tau in an Ultradian light-dark cycle. Journal of Biological Rhythms.

[bib0011] Buysse D.J. (2014). Sleep health: Can we define it? Does it matter?. Sleep.

[bib0012] Buysse D.J., Reynolds C.F., Monk T.H., Berman S.R., Kupfer D.J. (1989). The Pittsburgh sleep quality index: A new instrument for psychiatric practice and research. Psychiatry Research.

[bib0013] Byrne J.E.M., Bullock B., Murray G. (2017). Development of a measure of sleep, circadian rhythms, and mood: The SCRAM questionnaire. Frontiers in Psychology.

[bib0014] Chakradeo P., Rasmussen H.E., Swanson G.R., Swanson B., Fogg L.F., Bishehsari F., Burgess H.J., Keshavarzian A. (2022). Psychometric testing of a food timing questionnaire and food timing screener. Current Developments in Nutrition.

[bib0015] Chaput J.-P., Dutil C., Featherstone R., Ross R., Giangregorio L., Saunders T.J., Janssen I., Poitras V.J., Kho M.E., Ross-White A., Zankar S., Carrier J. (2020). Sleep timing, sleep consistency, and health in adults: A systematic review. Applied Physiology, Nutrition, and Metabolism = Physiologie Appliquee, Nutrition et Metabolisme.

[bib0016] Charvet C., Boutron I., Morvan Y., Le Berre C., Touboul S., Gaillard R., Fried E., Chevance A. (2022). How to measure mental pain: A systematic review assessing measures of mental pain. Evidence-Based Practice in Mental Health.

[bib0017] Chellappa S.L. (2021). Individual differences in light sensitivity affect sleep and circadian rhythms. Sleep.

[bib0018] Chrobak A.A., Krupa A., Dudek D., Siwek M. (2021). How soft are neurological soft signs? Content overlap analysis of 71 symptoms among seven most commonly used neurological soft signs scales. Journal of Psychiatric Research.

[bib0019] Chrobak A.A., Siwek M., Dudek D., Rybakowski J.K. (2018). Content overlap analysis of 64 (hypo)mania symptoms among seven common rating scales. International Journal of Methods in Psychiatric Research.

[bib0020] Coelho J., Montagni I., Micoulaud-Franchi J.-A., Taillard J., Philip P., Plancoulaine S., Tzourio C. (2024). Why circadian rhythmicity matters: Associations between sleep irregularity and mental health conditions during the Covid-19 health crisis. The World Journal of Biological Psychiatry.

[bib0021] Coelho J., Sanchez-Ortuño M.M., Martin V.P., Gauld C., Richaud A., Lopez R., Pelou M., Abi-Saab P., Philip P., Geoffroy P.-A., Palagini L., Micoulaud-Franchi J.-A. (2023). Content analysis of insomnia questionnaires: A step to better evaluate the complex and multifaceted construct of insomnia disorder. Psychiatry Research.

[bib0022] Czeisler C.A., Duffy J.F., Shanahan T.L., Brown E.N., Mitchell J.F., Rimmer D.W., Ronda J.M., Silva E.J., Allan J.S., Emens J.S., Dijk D.J., Kronauer R.E. (1999). Stability, precision, and near-24-hour period of the human circadian pacemaker. Science (New York, N.Y.).

[bib0023] Deibel S.H., Lewis L.M., Cleary J., Cassell T.T.S., Skinner D.M., Thorpe C.M. (2022). Unpredictable mealtimes rather than social jetlag affects acquisition and retention of hippocampal dependent memory. Behavioural Processes.

[bib0024] Di Milia L., Adan A., Natale V., Randler C. (2013). Reviewing the psychometric properties of contemporary circadian typology measures. Chronobiology International.

[bib0025] Di Milia L., Barnes-Farrell J.L., Laguerre R., Folkard S. (2024). The association between vigour and flexibility with injury and alertness during shift work. Chronobiology International.

[bib0026] Di Milia L., Folkard S. (2021). More than morningness: The effect of circadian rhythm amplitude and stability on resilience, coping, and sleep duration. Frontiers in Psychology.

[bib0027] Di Milia L., Folkard S., Hill J., Walker C. (2011). A psychometric assessment of the Circadian amplitude and phase scale. Chronobiology International.

[bib0028] Di Milia L., Smith P.A., Folkard S. (2004). Refining the psychometric properties of the circadian type inventory. Personality and Individual Differences*.*.

[bib0029] Dijk D.-J., Duffy J.F. (2020). Novel approaches for assessing circadian rhythmicity in humans: A review. Journal of Biological Rhythms.

[bib0030] Dosseville F., Laborde S., Lericollais R. (2013). Validation of a chronotype questionnaire including an amplitude dimension. Chronobiology International.

[bib0031] Dzierzewski J.M., Donovan E.K., Sabet S.M. (2021). The sleep regularity questionnaire: Development and initial validation. Sleep Medicine.

[bib0032] Evans J.D. (1996).

[bib0033] Fischer D., Klerman E.B., Phillips A.J.K. (2021). Measuring sleep regularity: Theoretical properties and practical usage of existing metrics. Sleep.

[bib0034] Fishbein A.B., Knutson K.L., Zee P.C. (2021). Circadian disruption and human health. The Journal of Clinical Investigation.

[bib0035] Flo E., Pallesen S., Magerøy N., Moen B.E., Grønli J., Hilde Nordhus I., Bjorvatn B. (2012). Shift work disorder in nurses – Assessment, prevalence and related health problems. PloS One.

[bib0036] Folkard S., Lombardi D.A., Spencer M.B. (2006). Estimating the circadian rhythm in the risk of occupational injuries and accidents. Chronobiology International.

[bib0037] Folkard S., Monk T.H., Lobban M.C. (1979). Towards a predictive test of adjustment to shift work. Ergonomics.

[bib0038] Fried E.I. (2017). The 52 symptoms of major depression: Lack of content overlap among seven common depression scales. Journal of Affective Disorders.

[bib0039] Fried E.I., Flake J.K., Robinaugh D.J. (2022). Revisiting the theoretical and methodological foundations of depression measurement. Nature Reviews Psychology.

[bib0040] Gauld C., Baillieul S., Martin V.P., Richaud A., Lopez R., Pelou M., Micoulaud-Franchi J.-A. (2024). Symptom content analysis of OSA questionnaires: Time to identify and improve relevance of diversity of OSA symptoms?. Journal of Clinical Sleep Medicine.

[bib0041] Gauld C., Lopez R., Geoffroy P.A., Morin C.M., Guichard K., Giroux É., Dauvilliers Y., Dumas G., Philip P., Micoulaud-Franchi J.-A. (2021). A systematic analysis of ICSD-3 diagnostic criteria and proposal for further structured iteration. Sleep Medicine Reviews.

[bib0042] Gauld C., Martin V.P., Richaud A., Baillieul S., Vicente L., Perromat J.-L., Zreik I., Taillard J., Geoffroy P.A., Lopez R., Micoulaud-Franchi J.-A. (2023). Systematic item content and overlap analysis of self-reported multiple sleep disorder screening questionnaires in adults. Journal of Clinical Medicine.

[bib0043] Gauld C., Ouazzani K., Micoulaud-Franchi J.-A. (2020). To split or to lump? Classifying the central disorders of hypersomnolence: Sleep split requires epistemological tools and systematic data-driven conceptual analysis. Sleep.

[bib0044] Gauld C., Wakefield J.C., Micoulaud-Franchi J.-A. (2025). Proposing a definition for sleep disorders: An epistemological review. Sleep Medicine Reviews.

[bib0045] Gubin D., Weinert D., Stefani O., Otsuka K., Borisenkov M., Cornelissen G. (2025). Wearables in chronomedicine and interpretation of circadian health. Diagnostics.

[bib0046] Guichard K., Micoulaud -Franchi Jean-Arthur, Philip P., Taillard J. (2021). Sleep deprivation therapy to reset the circadian pacemaker in a non-24-hour sleep-wake disorder: A case report. Journal of Clinical Sleep Medicine.

[bib0047] Honkalampi K., Järvelin-Pasanen S., Tarvainen M.P., Saaranen T., Vauhkonen A., Kupari S., Perkiö-Mäkelä M., Räsänen K., Oksanen T. (2021). Heart rate variability and chronotype - A systematic review. Chronobiology International.

[bib87] Hood S., Amir S. (2017). The aging clock: circadian rhythms and later life. J. Clin. Invest..

[bib0048] Horne J.A., Ostberg O. (1976). A self-assessment questionnaire to determine morningness-eveningness in human circadian rhythms. International Journal of Chronobiology.

[bib0049] Jafari Roodbandi A., Choobineh A., Daneshvar S. (2015). Relationship between circadian rhythm amplitude and stability with sleep quality and sleepiness among shift nurses and health care workers. International Journal of Occupational Safety and Ergonomics.

[bib0050] Juda M., Vetter C., Roenneberg T. (2013). The Munich ChronoType questionnaire for shift-workers (MCTQShift). Journal of Biological Rhythms.

[bib0051] Karstoft K.-I., Armour C. (2022). What we talk about when we talk about trauma: Content overlap and heterogeneity in the assessment of trauma exposure. Journal of Traumatic Stress.

[bib0052] Kim S., Lee H.-J. (2021). Validation of the 6-item evening chronotype Scale (ECS): A modified version of Composite Scale morningness. Chronobiology International.

[bib0053] Kobayashi Frisk M., Fagman E., Arvidsson D., Ekblom Ö., Börjesson M., Bergström G., Zou D. (2024). Eveningness is associated with coronary artery calcification in a middle-aged Swedish population. Sleep Medicine.

[bib0054] LaBuzetta J.N., Malhotra A., Zee P.C., Maas M.B. (2022). Optimizing sleep and circadian health in the NeuroICU. Current Treatment Options in Neurology.

[bib0055] Levandovski R., Sasso E., Hidalgo M.P. (2013). Chronotype: A review of the advances, limits and applicability of the main instruments used in the literature to assess human phenotype. Trends in Psychiatry and Psychotherapy.

[bib0056] Morgenthaler T.I., Lee-Chiong T., Alessi C., Friedman L., Aurora R.N., Boehlecke B., Brown T., Chesson A.L., Kapur V., Maganti R., Owens J., Pancer J., Swick T.J., Zak R. (2007). Practice parameters for the clinical evaluation and treatment of circadian rhythm sleep disorders. Sleep.

[bib0057] Ogińska H. (2011). Can you feel the rhythm? A short questionnaire to describe two dimensions of chronotype. Personality and Individual Differences.

[bib0058] Ottoni G.L., Antoniolli E., Lara D.R. (2011). The Circadian Energy Scale (CIRENS): Two simple questions for a reliable chronotype measurement based on energy. Chronobiology International.

[bib0059] Pandi-Perumal S.R., Smits M., Spence W., Srinivasan V., Cardinali D.P., Lowe A.D., Kayumov L. (2007). Dim light melatonin onset (DLMO): A tool for the analysis of circadian phase in human sleep and chronobiological disorders. Progress in Neuro-psychopharmacology & Biological Psychiatry.

[bib0060] Partonen T. (2015). Chronotype and health outcomes. Current Sleep Medicine Reports.

[bib0061] Putilov A.A. (2017). Owls, larks, swifts, woodcocks and they are not alone: A historical review of methodology for multidimensional self-assessment of individual differences in sleep-wake pattern. Chronobiology International.

[bib0062] Putilov A.A., Sveshnikov D.S., Puchkova A.N., Dorokhov V.B., Bakaeva Z.B., Yakunina E.B., Starshinov Y.P., Torshin V.I., Alipov N.N., Sergeeva O.V., Trutneva E.A., Lapkin M.M., Lopatskaya Z.N., Budkevich R.O., Budkevich E.V., Dyakovich M.P., Donskaya O.G., Plusnin J.M., Delwiche B., Colomb C., Neu D., Mairesse O. (2021). Single-item chronotyping (SIC), a method to self-assess diurnal types by using 6 simple charts. Personality and Individual Differences.

[bib0063] Randler C., Díaz-Morales J.F., Rahafar A., Vollmer C. (2016). Morningness-eveningness and amplitude - development and validation of an improved composite scale to measure circadian preference and stability (MESSi). Chronobiology International.

[bib0064] Reid K.J. (2019). Assessment of circadian rhythms. Neurologic. Clinics..

[bib0065] Reinberg, A., Vieux, N., & Andlauer, P. (1981). (Eds.),Night and shift work: Biological and social aspects, First Edition. ed. Pergamon Press, Oxford.

[bib0066] Reinberg A., Vieux N., Ghata J., Chaumont A.J., Laporte A. (1978). Circadian rhythm amplitude and individual ability to adjust to shift work. Ergonomics.

[bib0067] Roenneberg T., Foster R.G., Klerman E.B. (2022). The Circadian System, sleep, and the health/disease balance – A conceptual review. Journal of Sleep Research.

[bib0068] Roenneberg T., Pilz L.K., Zerbini G., Winnebeck E.C. (2019). Chronotype and social jetlag: A (self-) critical review. Biology.

[bib0069] Roenneberg T., Wirz-Justice A., Merrow M. (2003). Life between clocks: Daily temporal patterns of human chronotypes. Journal of Biological Rhythms.

[bib0070] Sletten T.L., Weaver M.D., Foster R.G., Gozal D., Klerman E.B., Rajaratnam S.M.W., Roenneberg T., Takahashi J.S., Turek F.W., Vitiello M.V., Young M.W., Czeisler C.A. (2023). The importance of sleep regularity: A consensus statement of the National Sleep Foundation sleep timing and variability panel. Sleep Health.

[bib0071] Smith C.S., Reilly C., Midkiff K. (1989). Evaluation of three circadian rhythm questionnaires with suggestions for an improved measure of morningness. The Journal of Applied Psychology.

[bib0072] Steele T.A., St Louis E.K., Videnovic A., Auger R.R. (2021). Circadian rhythm Sleep–Wake Disorders: A contemporary review of neurobiology, treatment, and dysregulation in neurodegenerative disease. Neurotherapeutics : The Journal of the American Society for Experimental NeuroTherapeutics.

[bib0073] Taillard J., Mullens E. (2018). Les outils validés pour le diagnostic des troubles du rythme circadien veille-sommeil (TRCVS) chez les adultes et enfants. Presse Médicale, Dossier thématique. L'horloge Biologique et ses Implications dans les Pathologies du Sommeil.

[bib0074] Taillard J., Philip P., Chastang J.F., Diefenbach K., Bioulac B. (2001). Is self-reported morbidity related to the circadian clock?. Journal of Biological Rhythms.

[bib0075] Taillard J., Sagaspe P., Philip P., Bioulac S. (2021). Sleep timing, chronotype and social jetlag: Impact on cognitive abilities and psychiatric disorders. Biochemical Pharmacology.

[bib0076] Torsvall L., Akerstedt T. (1980). A diurnal type scale. Construction, consistency and validation in shift work. Scandinavian Journal of Work, Environment & Health.

[bib0077] Van Dongen H.P.A., Dinges D.F. (2003). Investigating the interaction between the homeostatic and circadian processes of sleep-wake regulation for the prediction of waking neurobehavioural performance. Journal of Sleep Research.

[bib0078] Vetter C. (2020). Circadian disruption: What do we actually mean?. The European Journal of Neuroscience.

[bib0079] Visontay R., Sunderland M., Grisham J., Slade T. (2019). Content overlap between youth OCD scales: Heterogeneity among symptoms probed and implications. Journal of Obsessive-Compulsive and Related Disorders.

[bib0080] Vitale J.A., Weydahl A. (2017). Chronotype, physical activity, and sport performance: A systematic review. Sports Medicine New Zealand.

[bib0081] Vitiello M.V., Smallwood R.G., Avery D.H., Pascualy R.A., Martin D.C., Prinz P.N. (1986). Circadian temperature rhythms in young adult and aged men. Neurobiology of Aging.

[bib0082] Wakefield J.C. (1992). Disorder as harmful dysfunction: A conceptual critique of DSM-III-R's definition of mental disorder. Psychological Review.

[bib0083] Wickwire E.M., Geiger-Brown J., Scharf S.M., Drake C.L. (2017). Shift work and Shift work sleep disorder. Chest.

[bib0084] Windred D.P., Burns A.C., Lane J.M., Saxena R., Rutter M.K., Cain S.W., Phillips A.J.K. (2024). Sleep regularity is a stronger predictor of mortality risk than sleep duration: A prospective cohort study. Sleep.

[bib0085] Zare R., Choobineh A., Keshavarzi S. (2017). Association of amplitude and stability of circadian rhythm, sleep quality, and occupational stress with sickness absence among a gas company employees-A cross sectional study from Iran. Safety and Health at Work.

[bib0086] Zou H., Zhou H., Yan R., Yao Z., Lu Q. (2022). Chronotype, circadian rhythm, and psychiatric disorders: Recent evidence and potential mechanisms. Frontiers in Neuroscience.

